# Supplementary material for: Single Core Genome Sequencing for Detection of both Borrelia burgdorferi Sensu Lato and Relapsing Fever Borrelia Species
Source: Int J Environ Res Public Health. 2019 May 20;16(10):1779. doi: 10.3390/ijerph16101779 (PMC6571920; doi:10.3390/ijerph16101779)

Figure S1. Typical electropherogram showing a segment of diagnostic base-calling DNA-sequence of a 357-bp M1/M2 second PCR amplicon, sequenced with M2 primer, showing the underlined TTTTAG sequence complex characteristic of a *B. burgdorferi* 16S rRNA gene immediately downstream of the M1 primer binding site on the blood sample from a patient living in the United States. The underlined base "G" at position 54 indicates that this is not a *B. garinii* Bernie strain (See Figure 1, Sequence #2)

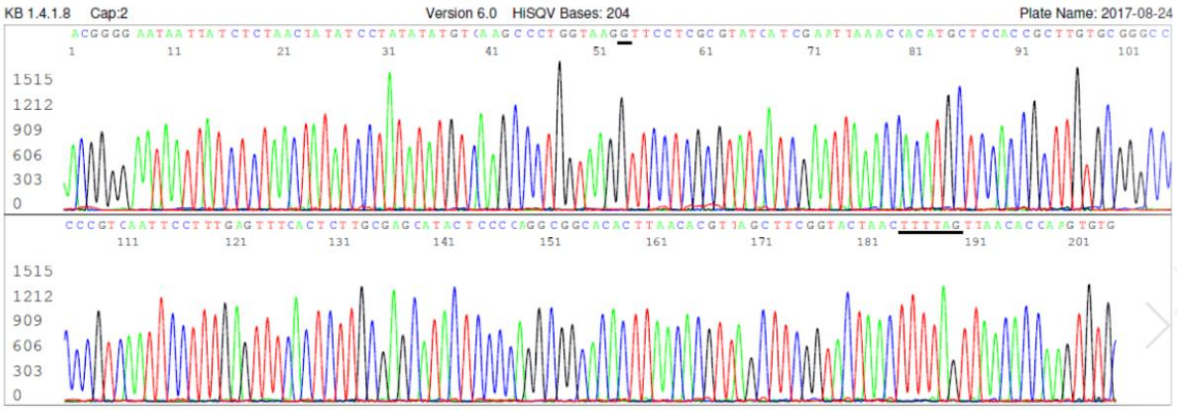

Figure S2. Reverse M1 primer sequencing of the 357-bp M1/M2 second PCR amplicon used to generate the DNA sequence in Figure S1, showing an underlined base "G" at position 201, indicating that this was a 16S rRNA gene sequence of *B. burgdorferi*, not *B. afzelii* (see Figure 1, Sequence #3, to read reverse complement). An underlined "C" at position 38 indicates that this was not a sequence of *B. garinii* Bernie strain. Based on single nucleotide polymorphisms, the less common *B. spielmanii* and *B. carolinensis* are also excluded. (see Figure 1, Sequences #2, #4 and #8 to read reverse complement).

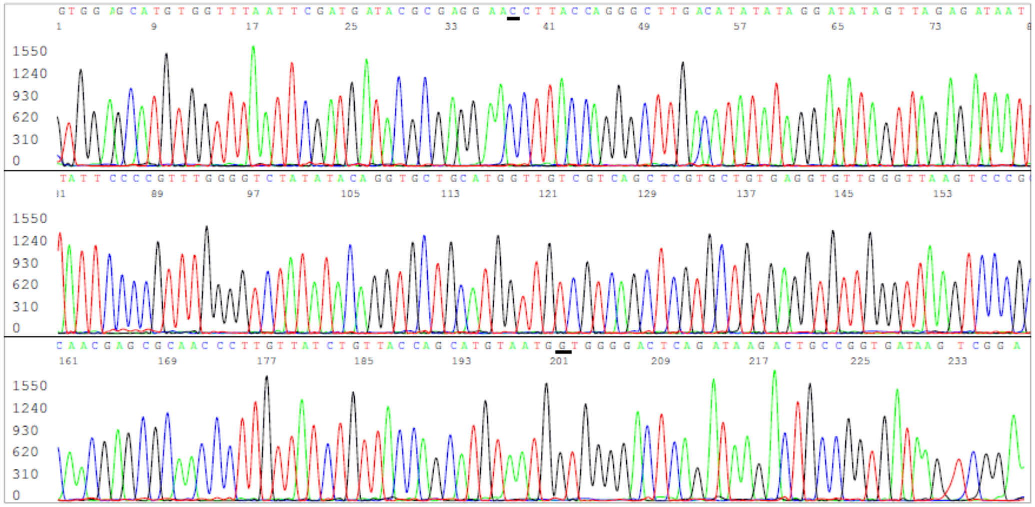

Figure S3. Typical sequence segment of the M1/M2 primer second PCR amplicon for molecular diagnosis of *B. garinii* Bernie strain in a tick collected in Portumna, Galway County, Ireland, using M2 sequencing primer (note the underlined base “A” at position 54, compared with the corresponding base “G” at position 54 in Figure S1 for *B. burgdorferi*)

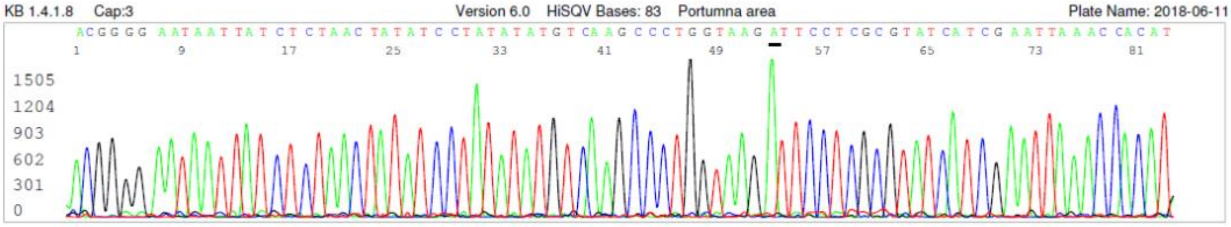

Figure S4. Typical sequence segment of the M1/M2 primer second PCR amplicon for molecular diagnosis of *B. garinii* Bernie strain in a tick collected in Portumna, Galway County, Ireland, using M1 as the sequencing primer (note the underlined base “T” at position 31, compared with the corresponding underlined base “C” at position 38 in Figure S2 for *B. burgdorferi*)

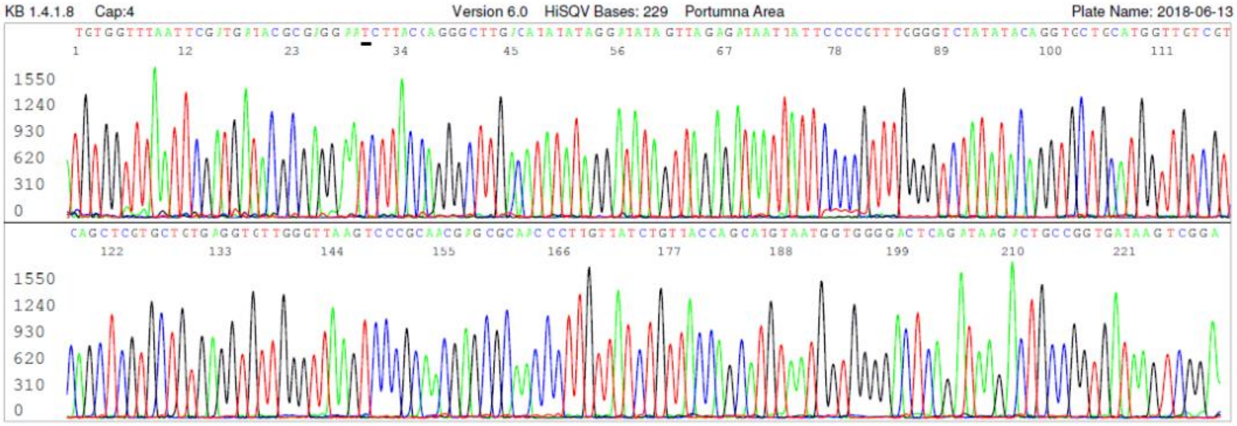

Figure S5. Typical segment of a diagnostic base-calling DNA-sequencing electropherogram from a 357-bp PCR amplicon, sequenced with M2 primer, showing the underlined TTTCAG sequence complex characteristic of *B. valaisiana* 16S rRNA gene immediately downstream of the M1 primer binding site (see Figure 1, Sequence #5) This sample was from a tick collected in Killarney, Kerry County, Ireland.

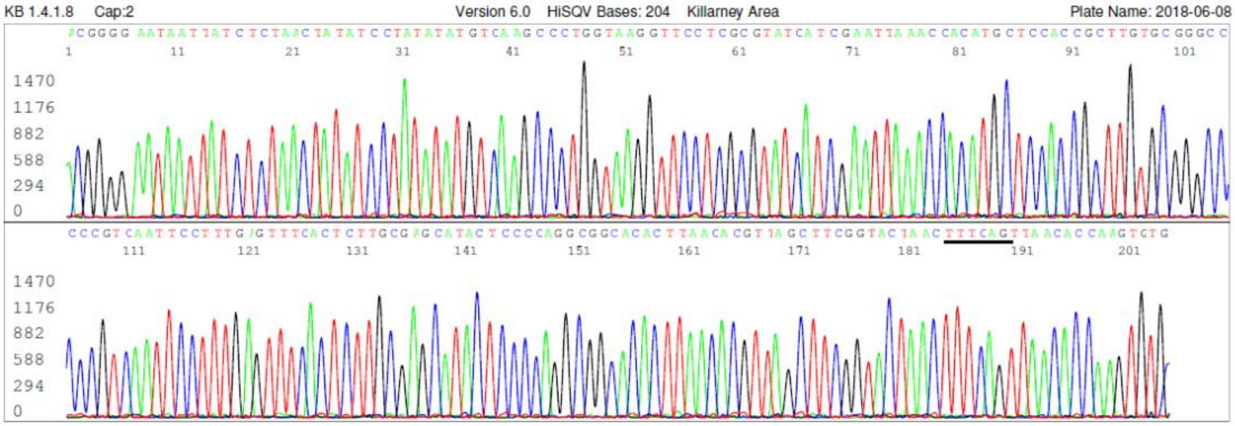

Figure S6. Typical segment of a diagnostic base-calling DNA-sequencing electropherogram from a 358-bp M1/M2 primer second PCR amplicon, sequenced with M2 primer, showing the underlined common relapsing fever borrelial 16S rRNA gene sequence complex CTTTCGA immediately downstream of the M1 primer binding site. The unique bases for *B. miyamotoi* in this segment were not marked (see Figure 1, Sequence #9). This sample was from a tick collected in Portumna, Galway County, Ireland. The underlined GTC sequence at position 11-13, in contrast to the sequence GTT at this position in Sequence #9, Figure 1, indicates that this strain of *B. miyamotoi* found in a tick in Ireland may be identical to Borrelia sp. LB-W100 often isolated in Europe (Sequence ID: AF529085)

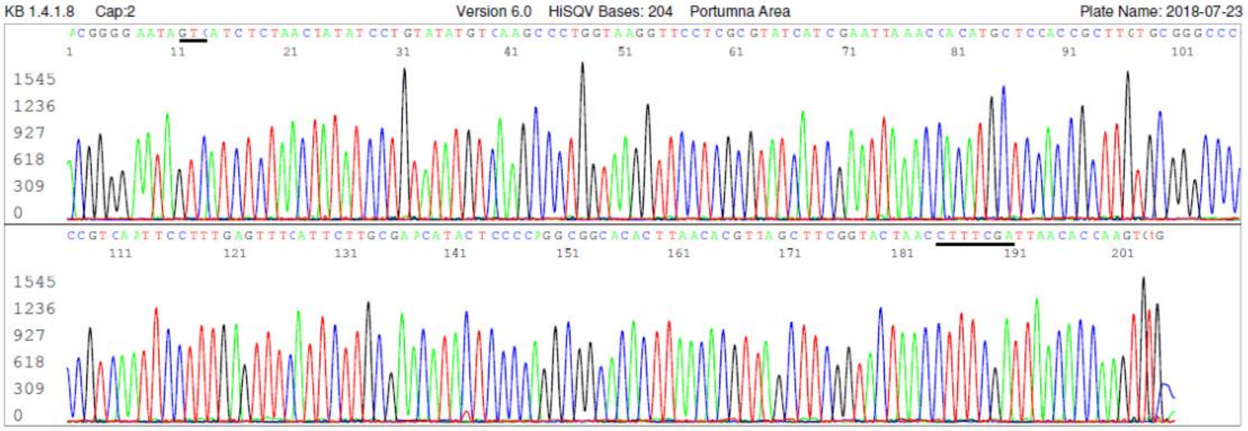

Figure S7 In contrast to the *B. miyamotoi* isolated from an Irish tick with a sequence shown in Figure S6, an isolate of *B. miyamotoi* from a sample of the serum repository panel accepted by the CDC as reference sera from "proven" Lyme disease patients was found to belong to *Borrelia miyamotoi* LB-2001 (Sequence ID: CP006647). The sequence of the M1/M2 primer second PCR amplicon with M1 sequencing primer was reported to the CDC on November 21, 2013 as follows. (Note the sequence AAC underlined in position 169-171 is the reverse complement of GTT).

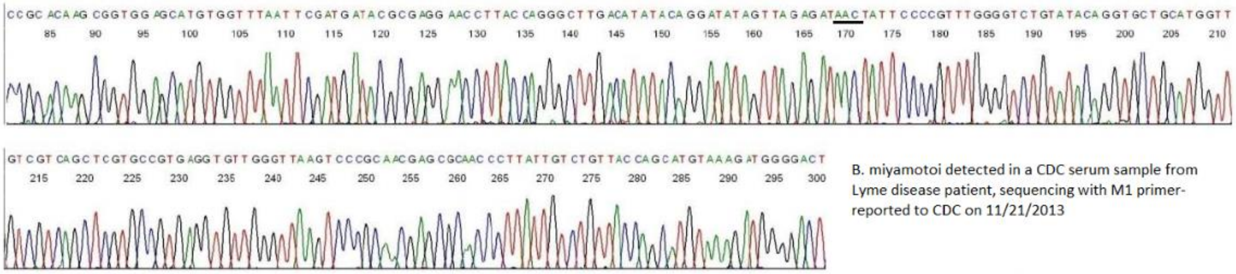

Figure S8. Sequence of the M1/M2 primer second PCR amplicon of the CDC serum sample (Figure S7) with M2 sequencing primer, showing a reverse complementary sequence of a strain of *B. miyamotoi* LB 2001 infecting a patient who was diagnosed as having Lyme disease (Note the underlined sequence GTT at position 22-24 compared to the underlined sequence GTC at position 11-13 in Figure S6 for the *B. miyamotoi* detected in a tick collected in Ireland).

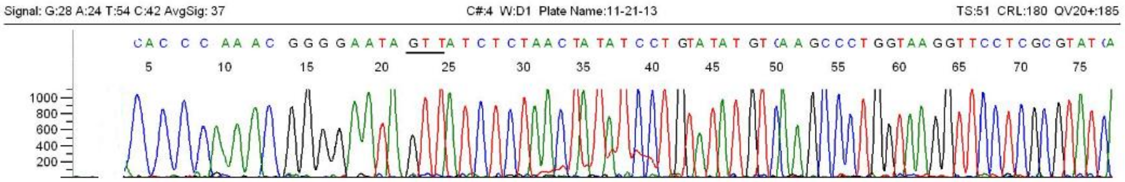

Figure S9. Blood sample from a patient with persistent co-infection by *B. burgdorferi* and *B. miyamotoi*. This was M2 primer sequencing of an M1/M2 primer second PCR amplicon. The black arrows indicate 3 superimposed base calling double peaks, C/T at position 211, G/A at 220 and T/C at 267 before the frameshift indels following peak 271 due to a one-nucleotide gap of the *B. burgdorferi* sequence in this segment (see Figure 1, compare Sequence #1 and Sequence #9), representing a feature characteristic of two overlapped sequences derived from these two borreliae. The underlined sequence A/GTT at position 94-96 indicates that the strain of *B. miyamotoi* in this mixed infection was *B. miyamotoi* LB 2001, not Borrelia sp. LB-W100 (see Figure S6, underlined sequence GTC).

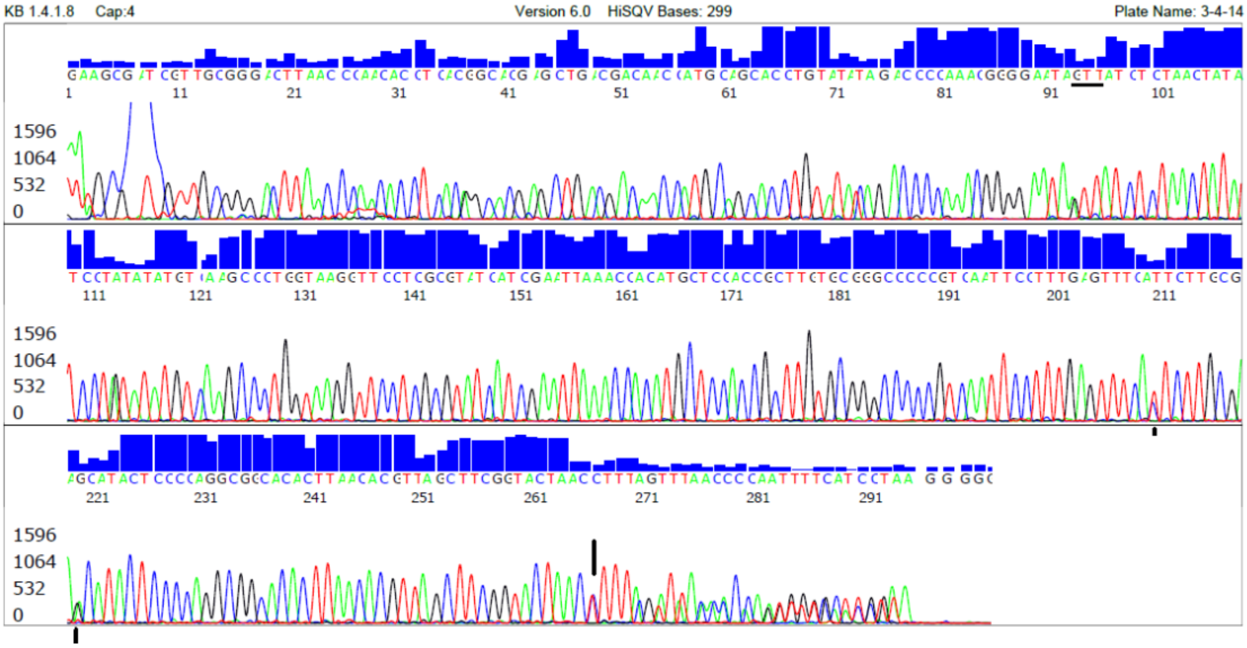

Figure S10. M2 primer sequencing segment of an M1/M2 primer second PCR amplicon derived from the DNA extract of a tick removed from the skin bite of a person living in Connecticut, U.S.A., showing a mixed infection by *B. burgdorferi* and *B. miyamotoi* with two overlapped sequences, similar to that illustrated in Figure S9. Again, the *B. miyamotoi* in the mixture was strain LB 2001, not Borrelia sp. LB-W100 as shown in the underlined sequence G/ATT at position 75-77. Note frameshift indels sequence after peak 252.

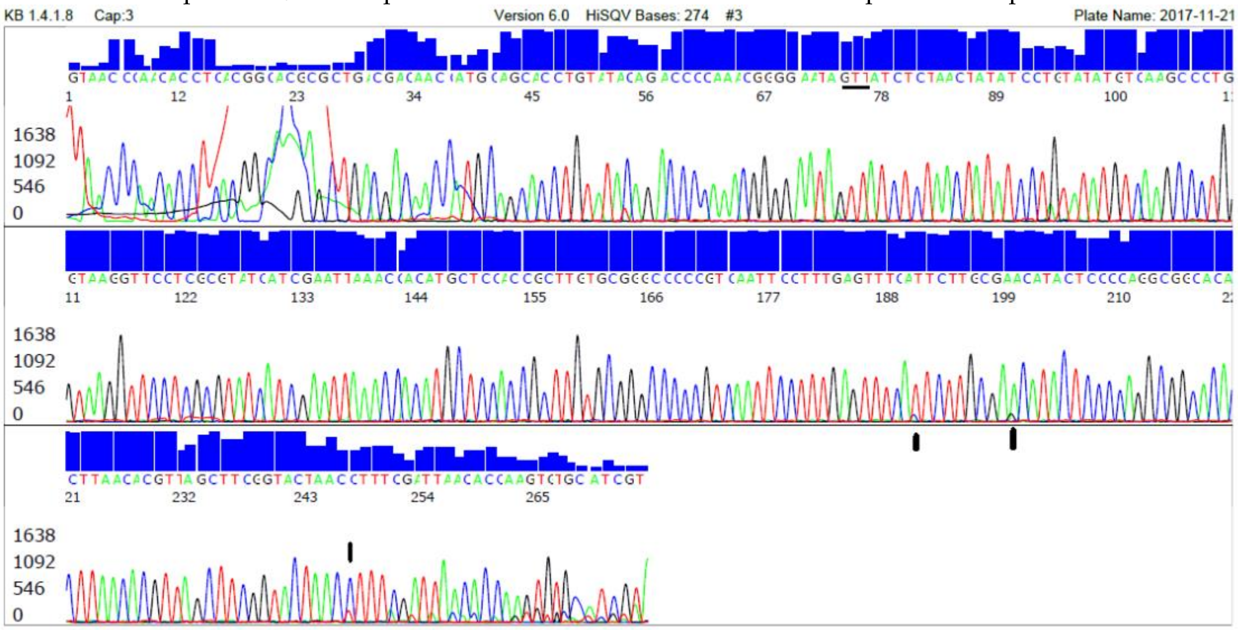

Figure S11. M2 primer 16S rRNA gene sequencing of the M1/M2 primer second PCR products of the DNA extract from a pure culture of *B. coriaceae* (ATCC 43381). Note the underlined common relapsing fever borrelial 16S rRNA gene sequence complex CTTTCGA at position 184-190 immediately downstream of the M1 primer site and the unique underlined base "T" at position 57 for *B. coriaceae* (see Figure 1, Sequence #11).

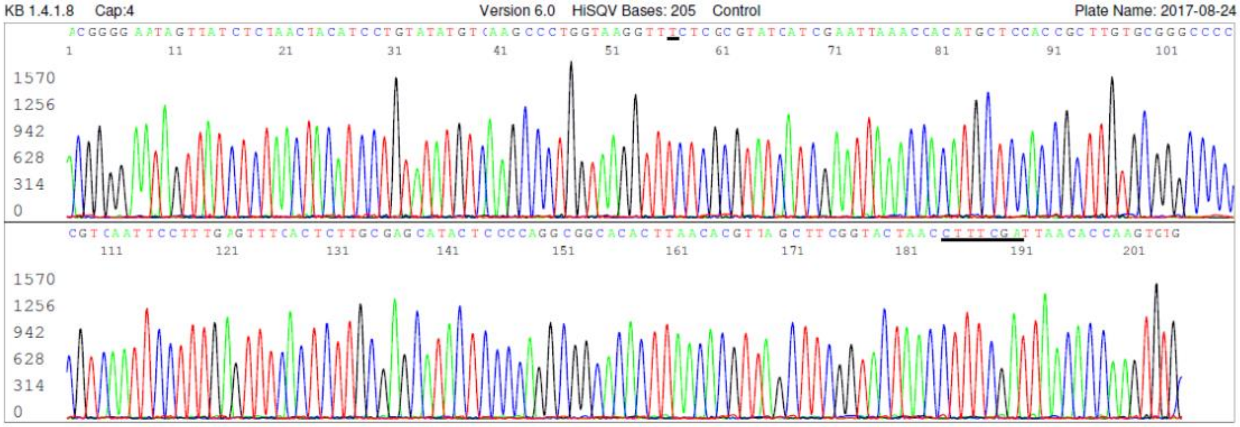

Figure S12. Segment of a 282-base 16S rRNA gene sequence defined by the Bg5 and Bg6 primers and generated with a Bg6 sequencing primer showing DNA of *B. burgdorferi* sensu lato from a tick collected in Connecticut. The sequence is that of a strain of *B. burgdorferi*, not *B. garinii* because of its unique underlined base "G" at position 63 (see Figure 2, Sequence #1)

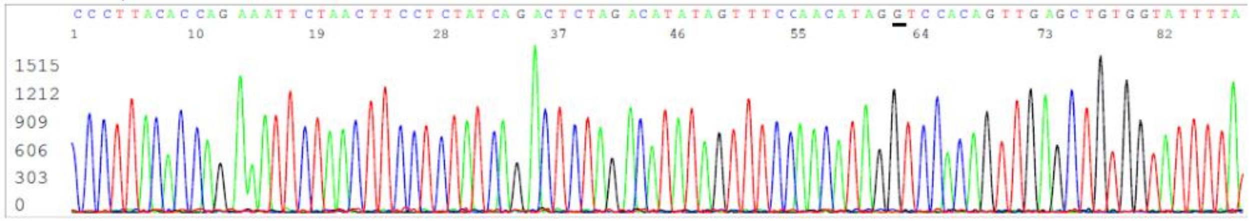

Figure S13. Segment of a 282-base 16S rRNA gene sequence defined by the Bg5 and Bg6 primers and generated with a Bg6 sequencing primer on the DNA extract of a tick collected in Portumna, Galway County, Ireland. The underlined base "T" at position 63 indicates that it was a strain of *B. garinii* (compare this base "T" with the underlined base "G" at position 63 in Figure S12 and see Figure 2, Sequences #1 and #3). Single nucleotide polymorphisms in this 282 -base sequence also indicated that this was a *B. garinii* Strain BgVir (Sequence ID: CP003151), not *B. garinii* Strain T25 (Sequence ID: AB035388), and not *B. garinii* Strain Bernie (Sequence ID: D89900).

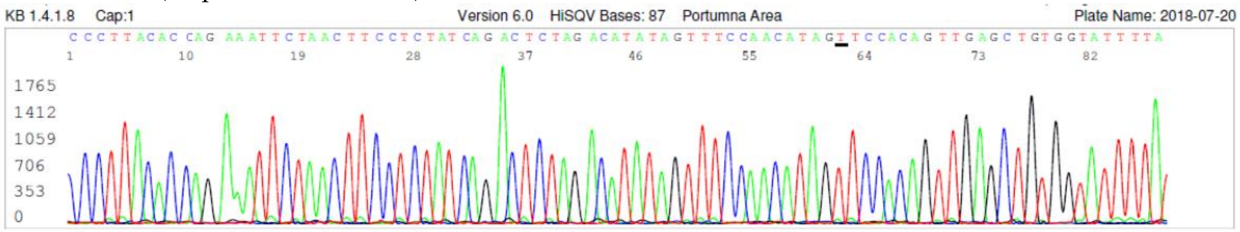

Figure S14. As illustrated in the five electropherograms pasted below, Sanger sequencing of the second (same-nested) PCR products of the NYS Lyme proficiency test samples #14, #15, #17, #18 and #20 (see Figure 3, lanes 14, 15, 17, 18 and 20) showed that the PCR products derived from samples NYS-15 and NYS-20 both consisted of DNA typical of a segment of *B. burgdorferi* sensu lato 16S rRNA gene sequence (Figure 1). The PCR product bands observed in lanes 14, 17 and 18 resulted from non-target PCR amplification in the absence of a target template. The M2 sequencing primer was not able to generate one DNA sequence on sample NYS-14 for the computer program to perform the function of base calling although the non-target PCR products generated on this sample migrated at roughly the same speed as the borrelia DNA to form a band of similar size at gel electrophoresis. The PCR products shown in lanes 17 and 18 were those of human genomic DNA as a result of amplification of non-target DNA with partial complementary sequence match with the PCR primers. Note: On NYS-17 sample sequence the M1 PCR primer annealed to a human genomic DNA segment with 11 matched nucleotides (underlined). On NYS-18 sample sequence the M1 PCR primer annealed to a human genomic DNA segment at position 152-160 (9 nucleotides underlined).

Biosystems

S/N G:4 A:3 T:5 C:4 | NYS-14 | Dec 21,2017 03:25PM, EST
KB.bcp | KB_3130_POP7_BDTv1.mob | Dec 21,2017 03:37PM, EST
KB 1.4.1.8 Cap:4 | Pts 2876 to 4686 Pk1 Loc:1599 | Spacing:11.24 Pts/Panel1100
Version 6.0 HiSQV Bases: 172 Lyme | Plate Name: 2017-12-21

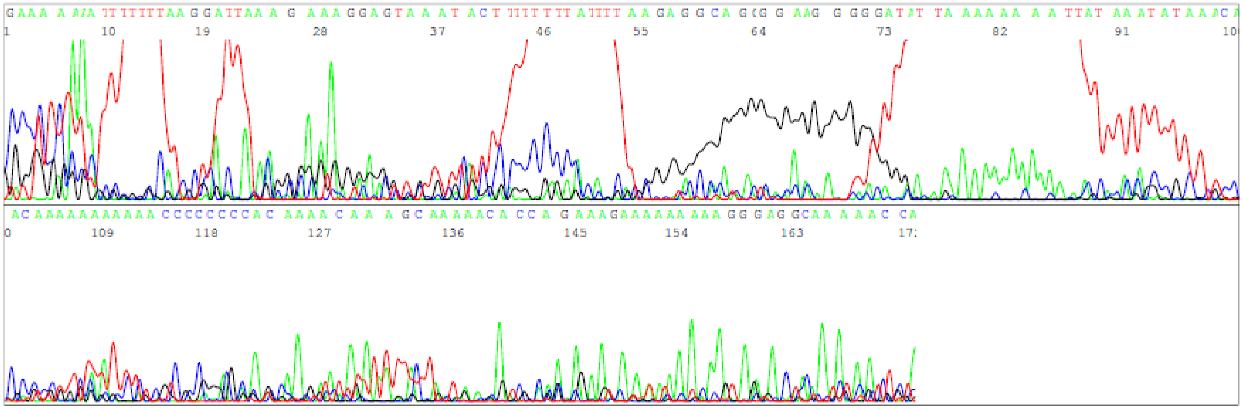

Biosystems

S/N G:114 A:108 T:173 C:204 | NYS-15 | Dec 21,2017 03:57PM, EST
KB.bcp | KB_3130_POP7_BDTv1.mob | Dec 21,2017 04:09PM, EST
KB 1.4.1.8 Cap:1 | Pts 2445 to 5495 Pk1 Loc:1869 | Spacing:11.41 Pts/Panel1300
Version 6.0 HiSQV Bases: 273 Lyme | Plate Name: 2017-12-21

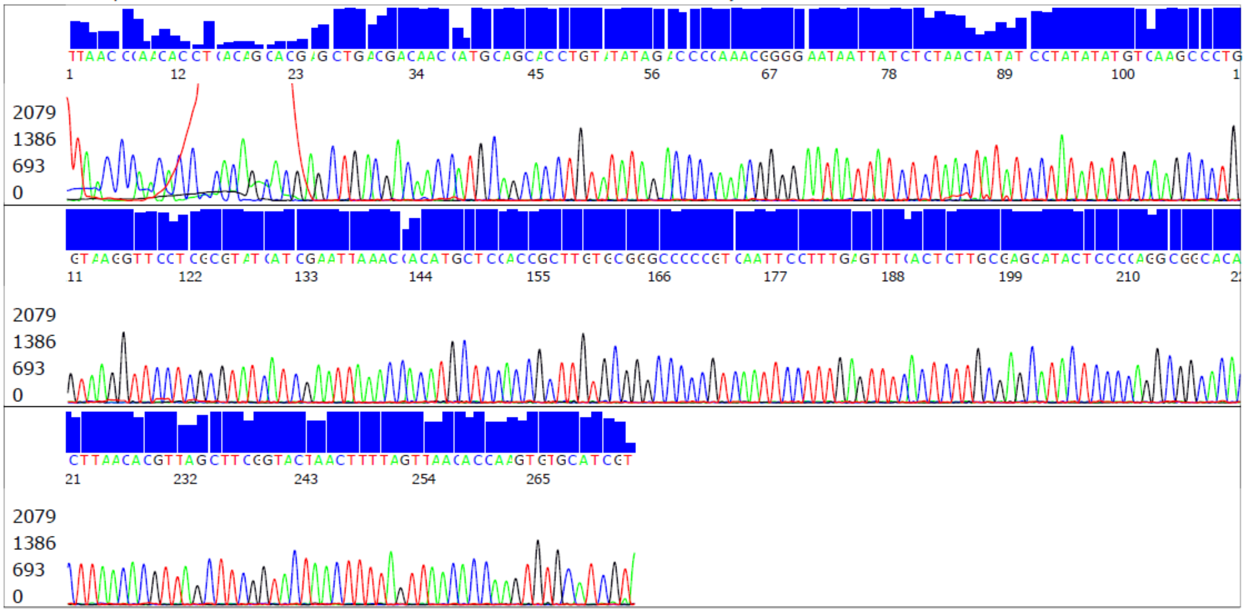

Biosystems

S/N G:516 A:592 T:747 C:955 | NYS-17 | Dec 21,2017 03:57PM, EST
KB.bcp | KB_3130_POP7_BDTv1.mob | Dec 21,2017 04:09PM, EST
KB 1.4.1.8 Cap:2 | Pts 2304 to 3608 Pk1 Loc:1900 | Spacing:11.3 Pts/Panel1300
Version 6.0 HiSQV Bases: 122 Lyme | Plate Name: 2017-12-21

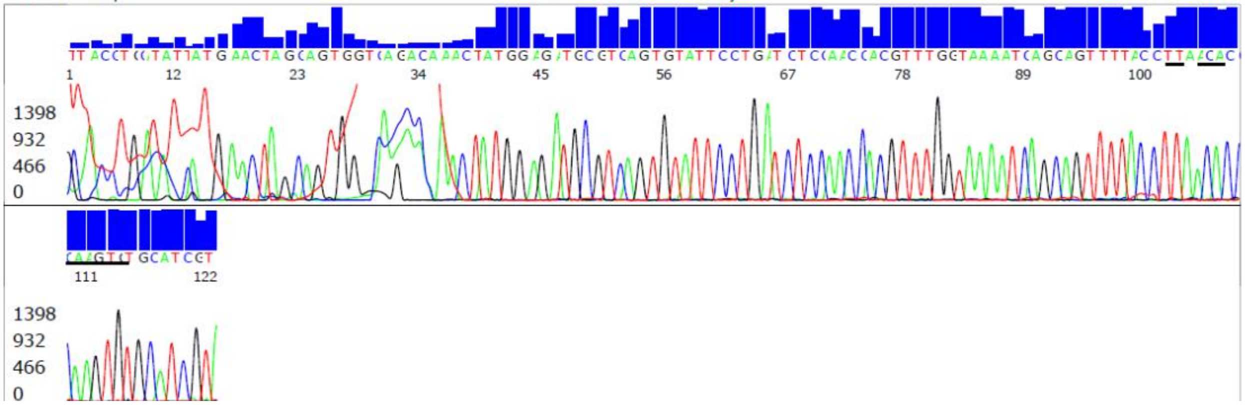

Submission of NYS-17 sequence to the GenBank for BLAST alignment analysis confirmed that it is a segment of human genomic DNA within the "Homo sapiens chromosome 20" (see GenBank report below) which was amplified by the M1 and M2 primers in a human blood sample negative for borrelia DNA.

NYS sample # 17

Homo sapiens protein tyrosine phosphatase, receptor type T (PTPRT), RefSeqGene on chromosome 20

Sequence ID: NG_033880.1Length: 1124166Number of Matches: 1

Related Information

Map Viewer-aligned genomic context

Range 1: 844963 to 845037GenBankGraphics Next Match Previous Match

Alignment statistics for match #1

Score	Expect	Identities	Gaps	Strand
128 bits(69)	2e-26	73/75(97%)	0/75(0%)	Plus/Minus

```
Query  1       ATGGAGATGCGTCAGTGTATTCCTGATCTCCAACCACGTTTGGTAAAATCAGCAGTTTTA  60
               ||||||||||||||||||||||||||||||||||||||||||||||||||||||||||||
Sbjct  845037  ATGGAGATGCGTCAGTGTATTCCTGATCTCCAACCACGTTTGGTAAAATCAGCAGTTTTA  844978

Query  61      CCTTAACACCAAGTG  75
               |||| ||| ||||||
Sbjct  844977  CCTTGACAGCAAGTG  844963
```

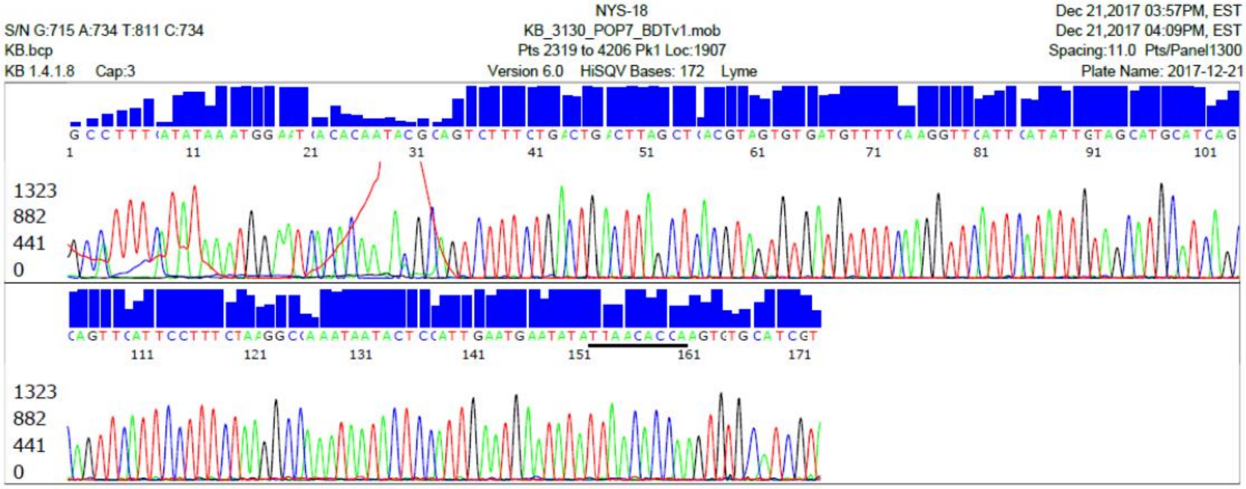

Submission of NYS-18 sequence to the GenBank for BLAST alignment analysis confirmed that it is a segment of human genomic DNA within the "Homo sapiens chromosome 8" (see GenBank report below) which was amplified by the M1 and M2 primers in a human blood sample negative for borrelia DNA.

NYS sample 18

Homo sapiens chromosome 8, clone RP11-90P5, complete sequence

Sequence ID: AC084024.17Length: 135404Number of Matches: 1

Related Information

Map Viewer-aligned genomic context

NewGenome Data Viewer-aligned genomic context

Range 1: 64240 to 64365GenBankGraphics Next Match Previous Match

Alignment statistics for match #1

Score	Expect	Identities	Gaps	Strand
233 bits(126)	7e-58	126/126(100%)	0/126(0%)	Plus/Minus

```
Query  1      TCTTTCTGACTGACTTAGCTCACGTAGTGTGATGTTTTCAAGGTTCATTCATATTGTAGC  60
              ||||||||||||||||||||||||||||||||||||||||||||||||||||||||||||
Sbjct  64365  TCTTTCTGACTGACTTAGCTCACGTAGTGTGATGTTTTCAAGGTTCATTCATATTGTAGC  64306

Query  61     ATGCATCAGCAGTTCATTCCTTTCTAAGGCCAAATAATACTCCATTGAATGAATATATTA  120
              ||||||||||||||||||||||||||||||||||||||||||||||||||||||||||||
Sbjct  64305  ATGCATCAGCAGTTCATTCCTTTCTAAGGCCAAATAATACTCCATTGAATGAATATATTA  64246

Query  121    ACACCA  126
              ||||||
Sbjct  64245  ACACCA  64240
```

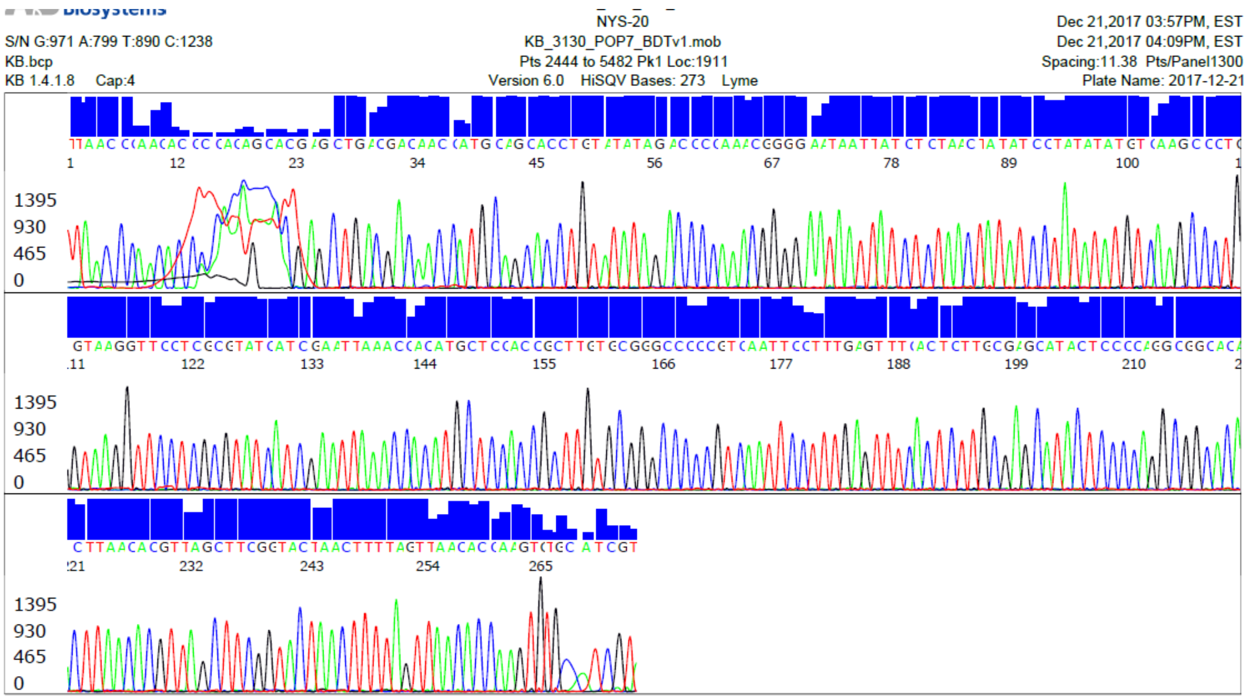

Figure S15. Segment of a 282 bp *B. miyamotoi* 16S rRNA gene sequence defined by Bg5 and Bg6 primers (see Figure 2). The sequencing primer was Bg6. The sample DNA used to initiate the PCR was from the same NH_4OH digestate of the tick collected in Portumna, Galway County, Ireland which was used to generate the DNA sequence presented in Figure S6. Comparing the sequence presented in Figure S6 and the sequence presented in Figure S15 showed that the PCR products amplified by the M1/M2 primers (Figure S6) were generated by a single target template, and were free of ambiguous base-calling peaks on the sequencing electropherogram. However, when the PCR products generated on the same sample by a pair of Bg5/Bg6 primers were sequenced, numerous ambiguous base-calling peaks appeared on the

electropherogram and the sequences “TT” at positions 4-6, 17-19 and 25-27 were misread as “TTT” by the computer due to interference by non-target DNA PCR products.

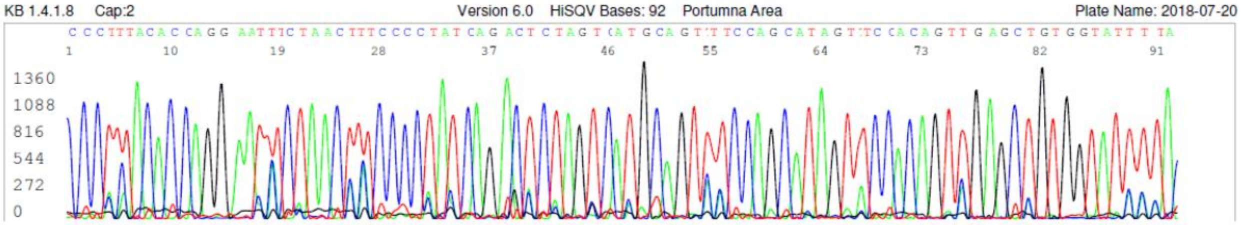

Figure S16. Segment of a 282 bp *B. coriaceae* 16S rRNA gene sequence defined by Bg5 and Bg6 primers generated in the same experiment described above (for Figure S15) as positive control, showing a unique base “C” at position 33 (see Figure 2, Sequence #11). Note the almost identical ambiguous base-calling peaks on Figure S16 as those shown in the Figure S15 electropherogram and the same sequences “TT” at positions 4-6, 17-19 and 25-27 being misread as “TTT” by the computer due to interference by non-target DNA PCR amplification. Since the nested PCR products used to generate the sequence in Figure S16 were those amplified from the DNA extract of a pure culture of *B. coriaceae* with a highly conserved relapsing fever borrelia 16S rRNA gene, the almost identical non-target DNA PCR products causing the ambiguous base-calling peaks in the electropherograms in both Figures S15 and S16 must have resulted from amplification of certain relapsing fever borrelia genomic DNA fragments by the Bg5 and Bg6 primers. As shown in Figures S12 and S13, the Bg5/Bg6 heminested PCR primer pair did not generate similar interfering non-target DNA products from the DNA extracts of *B. burgdorferi* sensu lato species.

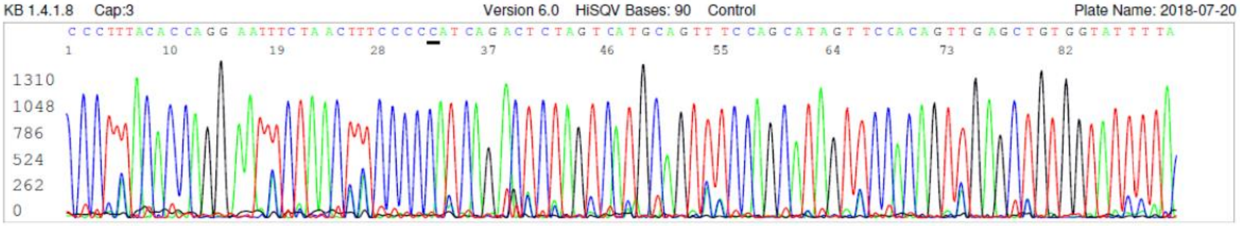

Supplement: Supplementary file 1 [file ijerph-16-01779-s001.pdf]
